# Evaluating the performance of selected military hospitals in Tehran in response to Covid‐19 pandemic: A cross‐sectional study

**DOI:** 10.1002/hsr2.2030

**Published:** 2024-04-10

**Authors:** Simintaj Sharififar, Maryam Moradi

**Affiliations:** ^1^ Department of Health in Disaster and Emergencies School of Nursing Aja University of Medical Sciences Tehran Iran; ^2^ Aja University of Medical Sciences Tehran Iran

**Keywords:** Covid‐19, hospital, pandemic, performance evaluation

## Abstract

**Background and Aims:**

The rapid spread of coronavirus disease 2019 (Covid‐19) led the need to admit a large number of infected people to hospitals in a short period of time, turning them into one of the most important responsive organizations. This study aims to evaluate the performance of selected military hospitals because they carried out a military operation in Tehran in response to the recent pandemic.

**Methods:**

This is a descriptive‐analytical study. The statistical population of this study consisted of military hospitals responding to Covid‐19 pandemic in Tehran. A checklist to evaluate the performance of hospitals in response to Covid‐19 pandemic (six areas, 23 sub‐areas and 152 items) was used as a data collection tool in this study. This tool had six domains, including risk management and planning, coordination and communication, infection prevention and control, diagnosis and treatment, education and training, and resource management.

**Results:**

The overall performance of selected hospitals was 63%, which indicated a good performance. The domain of coordination and communication obtained the lowest score.

**Conclusion:**

The investigated hospitals had good performance because they had a desirable access to resources. Periodic self‐assessment and accreditation is recommended to improve the performance of these hospitals.

## 1 INTRODUCTION

The occurrence of bioterrorist incidents and pandemics of newly emerging and re‐emerging diseases, which recently occurred in the world, highlight the necessity of preliminary preparations for all health and treatment centers.^1^, ^2^

As of January 2020, the world has been gripped by an outbreak of coronavirus disease 2019 (Covid‐19) that was soon announced as a pandemic by WHO, with more than 98 million cases and 2.1 million deaths reported (43 million cases and 1.143 million deaths within the 10 months of first reported case).^3^ Due to the rapid spread of Covid‐19, its high infection rate and mortality in severe cases, and also uncertainty in pharmacological treatment of patients, this disease was soon considered a great threat to human life and health.^4^ The outbreak of Covid‐19 as a public health emergency caused international concern, and hospitals needed strategies to manage their capacity, staff, and supplies to provide optimal care to patients.^5^ Meanwhile, hospitals and healthcare workers are directly affected by such incidents and crises.^6^, ^7^ As a place of care and treatment, hospitals are vulnerable to disasters and crisis. They are also among the institutions that need to continue operating after the onset of crisis, or have to increase their capacity in a short notice. Taking into account the speed and volume of damages that can be caused by biological events, unlike other incidents, it is not possible to rely on medical facilities and hospitals outside the affected area, so it is necessary for military hospitals in each region to be sufficiently prepared to respond to biological disasters.^8^

In recent years in Iran, the armed forces have played an effective role in responding to disasters. During these years, health centers and hospitals of the armed forces have been among the most responsive organizations in the health sector.^9^ Increasing the preparedness of military healthcare personnel to respond to disasters can promote optimal performance of military hospitals in future global health emergencies. The strategic document on health, relief and treatment of the armed forces in Iran states that the main mission of military hospitals is to provide relief and also transfer and treat the injured in unexpected incidents. Therefore, the preparedness of military hospitals in accidents and disasters is more important than other hospitals. In the meantime, military health and treatment centers, considering their special missions and the key role they play in responding to crisis, should have plans to control the crisis, reduce its effects (if it occurs), and make the necessary preparations for future accidents and disasters.^10^ Therefore, providing appropriate measures to minimize the effects of accidents is considered as one of the main missions of the health system.^11^ According to the recommendations of the health system in regard to planning, preparing, and responding to disasters, it is necessary to use the “all hazards” approach. However, in practice, this approach does not seem to be suitable for man‐made and technological disasters such as biological, chemical or nuclear accidents. Nevertheless, evaluating the performance of hospitals during crisis, especially crisis caused by biological agents (e.g., disease pandemics, both natural and intentional), is an issue that has been insufficiently addressed.^8^ Since hospitals face different conditions in the face of disasters and biological threats, evaluating their performance in such conditions requires a different mechanism (a scientific process based on the successful models used in the world). Therefore, this study was conducted with the aim of evaluating the performance of selected military hospitals in Tehran during the Covid‐19 pandemic.

## 2 METHOD

This is a descriptive‐analytical study that was conducted in 2019 with the aim of evaluating the performance of military hospitals in response to the Covid‐19 pandemic in Tehran. The statistical population was military hospitals. Sampling in this study was done by census method and included four army hospitals in Tehran city that are currently admitting patients with Covid‐19 virus or had a history of admitting such patients. The exclusion criteria included the unwillingness of hospitals to enter the study, and one hospital was excluded from this research. In this study, the checklist for evaluating the performance of hospitals during the Covid‐19 pandemic was used. The validity and reliability of this checklist have been measured in the study of Salarvand et al.^12^ This checklist was designed and developed during Covid‐19 pandemic, and its validation process started in February 2019. The construct validity of this tool has been measured based on two methods of convergent validity and known groups. The content validity ratio of this tool (92%) and its content validity index (79%) have also been calculated. The internal consistency of this tool was determined to be 0.98 using Cronbach's alpha test. This checklist consists of four parts: (1) the information of the evaluating team, (2) the demographic characteristics of the hospitals (location, number of beds, type of hospital in terms of generality and specialty, the number of patient admission, and the number of patients recovered or died from Covid‐19), (3) the performance of hospitals in managing patients during the past year, and (4) the main structure of the checklist with six main areas, 23 sub‐areas and 152 items. The performance evaluation indexes include six areas (risk management and planning, coordination and communication, infection prevention and control, diagnosis and treatment, education and training, and resource management) (Table 1). The scoring system in this tool is based on three‐option Likert scale (yes, to some extent, and no), which classifies hospitals in four categories of poor (152–228), average (228–304), good (304–380), and excellent (456–380) in terms of performance.

**Table 1:** Domain and subdomains of the checklist for evaluating the performance of hospitals in response to Covid‐19 pandemic.

Domain	Subdomain	Number of items	Score	Total number of items	Score
Risk management and planning	Incident command	5	5–15	16	16–48
	Planning	4	4–12		
	Concurrent events	3	3–9		
	Performance evaluation	4	4–12		
Coordination and communication	Internal and external coordination	4	4–12	11	11–33
	Risk communication	7	7–21		
Infection control and prevention	Infection control	15	15–45	25	25–75
	Quarantine and isolation	5	5–15		
	Decontamination	5	5–15		
Diagnosis and treatment	Diagnosis ability	12	12–24	29	29–87
	Diagnosis speed	3	3–9		
	Biological triage	3	3–9		
	Treatment	4	4–12		
	Clinical ethics	4	4–12		
	Psychological services	3	3–9		
Education and training	Staff training	8	8–24	11	11–33
	Client training	3	3–9		
Resource management	Human resources	18	18–54	60	60–180
	Supply and logistics	21	21–63		
	Physical resources	6	6–18		
	Increase capacity	7	7–21		
	Continuity of service	5	5–15		
	Funds	3	3–9		
Whole tool		152	152–456	152	152–456

### 2.1 Scoring

Considering the three‐option scale (yes, somewhat, and no) for the tool and its normative status, the linear transformation method based on the number 100 was proposed as a guide to evaluate scores and judge the tool's performance. The standard performance score of hospitals was calculated based on the linear transformation method as follows:

L.T = (NH − Nmin)/(Nmax − Nmin) × 100, where NH is the hospital's raw performance score, Nmin is the minimum score of the tool, Nmax is the maximum score of the tool and L.T is the standardized score of the hospital's performance (Table 1).

After completing the checklist for the selected hospitals, data description, including overall evaluation score and score of each domain, was determined based on demographic characteristics and evaluation scores. Mean, standard deviation, and frequency were used to analyze the data through SPSS‐24 software.

### 2.2 Ethical statement and consent to participate

The study was reviewed and approved by the Ethics Committee of Aja University of Medical Science (IR.AJAUMS.REC.1399.098). Attached to each questionnaire was a cover letter explaining what was expected of the respondents who had to sign, indicating their informed consent before they provided answers. In this way, the full understanding and the voluntary participation of the respondents was established. Throughout the research, confidentiality was respected and ensured.

## 3 RESULTS

The information related to the year 2019 was analyzed in the first part of the checklist and the performance of studied hospitals (the second part of the checklist) in March 2019 was evaluated.

**Figure 1:**
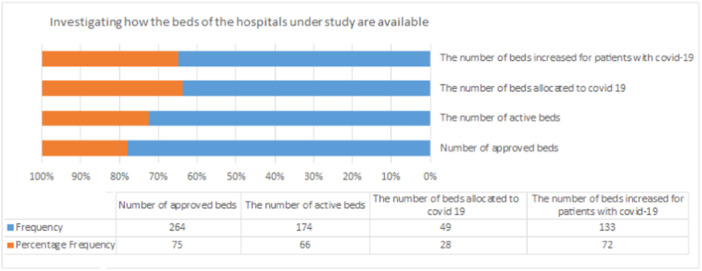
Status of beds in the selected hospitals.

The bed status in the studied hospitals was investigated in terms of the number of approved beds, active beds, the number of beds allocated to Covid‐19 patients, and the increased number of beds for Covid‐19 patients. Figure 1 shows that 72% of hospital beds are allocated to Covid‐19 patients.

The score of studied hospitals' safety is presented in Table 2, according to the latest evaluation by the FHSI tool. The mean accreditation score of the domain of risk management and planning was 88.7 in the selected hospitals.

**Table 2:** Safety score of selected hospitals in Tehran according to the latest FHSI assessment.

	Minimum	Maximum	Mean
Total	81.9	86.5	84.7
Structural	81.7	98.3	92.2
Nonstructural	71.6	82.1	75.3
Performance	82.3	99.1	90.1
Score of accreditation for the domain of risk management and planning			
Accreditation	75.7	100	88.7

Table 3 shows the type of activity and the score of accreditation in the studied hospitals. Accordingly, 33.3% of hospitals were classified as superior level one and 66.7% as level one. In this study, 33.3% of hospitals were classified as general, and 66.7% were classified as specialized (Table 3).

**Table 3:** Frequency of the studied hospitals' specialty.

Specialty	Frequency percentage	Frequency
General	33.3	33.3
Specialized	66.7	100
Accreditation score of the selected hospital		
Superior level one	33.3	33.3
Level one	66.7	100

**Figure 2:**
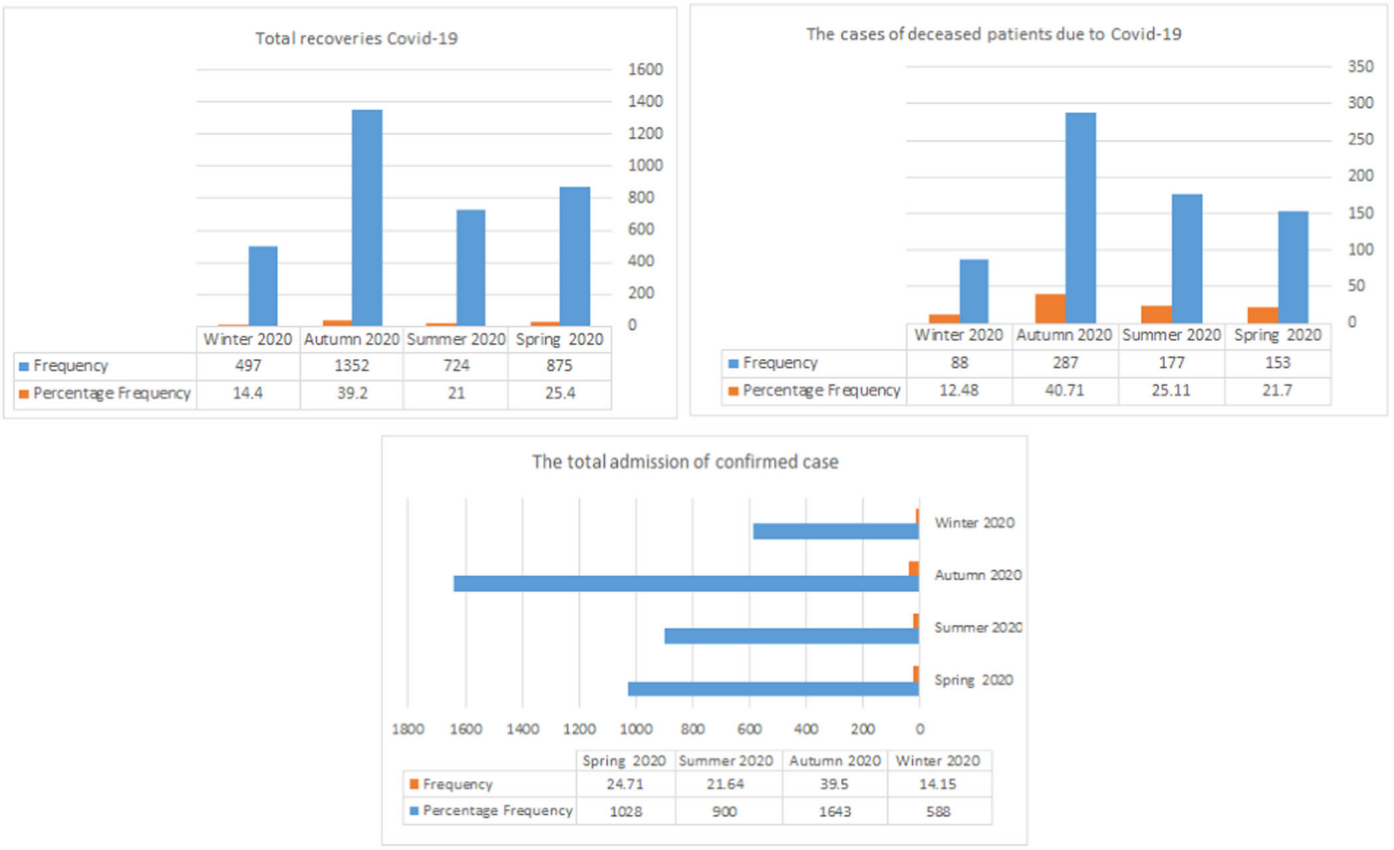
The performance of selected hospitals in terms of admitting patients with confirmed diagnosis of Covid‐19, patients recovered from Covid‐19, and deceased patients due to Covid‐19 in 2019.

The admission rate of patients with confirmed diagnosis, and the number of recovered and deceased patients from Covid‐19 in the studied hospitals showed that the highest admission of confirmed cases was related autumn season and the lowest was related to the winter season of 2019. The most recovered patients from the total number of patients admitted in 2019 were related to the winter season, and the most deaths occurred in the summer season. The lowest number of deaths among the total number of patients admitted in 2019 occurred in the winter and spring of 2019 (Figure 2).

**Figure 3:**
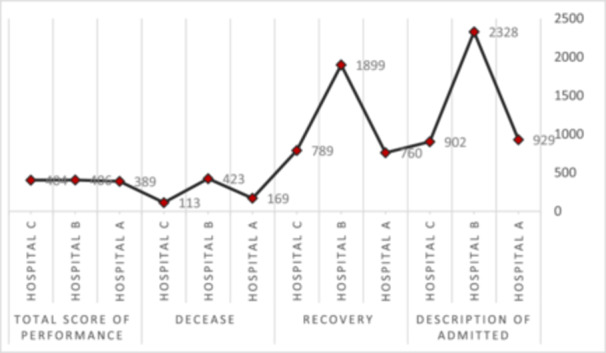
Performance, hospitalization of confirmed patients, patients recovered from Covid‐19, and deceased patients in each of the hospitals examined.

In the present study, each item was assigned a number according to the corresponding checklist in each hospital. In fact, the number of points in this study was not given by many people, and it is a single number, so the confidence interval, uncertainty, and the central and widely used indicator in this study are practically impossible. Hospitalization status of confirmed patients The number of recoveries and deaths due to Covid‐19 in each of the hospitals examined showed that the highest number of hospitalizations, confirmed cases, recoveries, and deaths due to Covid‐19 occurred in Hospital B and this hospital was the highest proven values showed results in this range (Figure 3).

Table 4 shows the score of performance evaluation in the studied hospitals as a whole and in each area, separately. These components include 6 areas, the highest mean of which is related to the areas of risk management and planning (93), and resource management (92). The lowest score of performance evaluation in the studied hospitals is related to the area of coordination and communication. Hospital B obtained the highest score in all areas except for the area of diagnosis and treatment. In the final ranking of the selected hospitals, hospitals B, C, and A ranked first, second, and third, respectively. In general, the performance of selected hospitals, based on the tools used, was evaluated at a good level (Table 4).

**Table 4:** Performance of the studied hospitals.

Domain		Mean	Performance
Risk management and planning	Hospital A	44.8	80
	Hospital B	48	100
	Hospital C	46.7	92
	Total	47	93
Communication and coordination	Hospital A	30	72
	Hospital B	31	82
	Hospital C	31	82
	Total	30.66	79
Infection control and prevention	Hospital A	67	68
	Hospital B	74	96
	Hospital C	73	92
	Total	71.33	85
Diagnosis and treatment	Hospital A	80	76
	Hospital B	81	79
	Hospital C	84	89
	Total	81.67	82
Education and training	Hospital A	30	72
	Hospital B	33	100
	Hospital C	33	100
	Total	32	90
Resource management	Hospital A	175	92
	Hospital B	176	93
	Hospital C	174	90
	Total	175	92
Total score of performance	Hospital A	389	56
	Hospital B	406	67
	Hospital C	404	66
Total		400	63

## 4 DISCUSSION

The main goal of this study was to evaluate the performance of selected military hospitals in response to Covid‐19 pandemic.

The performance of selected hospitals was evaluated in six domains of risk management and planning, coordination and communication, infection prevention and control, diagnosis and treatment, education and training, and resource management. The performance of selected hospitals was classified at a good level. The lowest score was related to the domain of coordination and communication and the highest score was related to the domain of risk management and planning. The overall performance score of the selected military hospitals was 63 out of 100, and their performance was classified at good. In the study of Mirzaei et al.^13^ (1400), the level of preparedness of hospitals affiliated to Yazd University of Medical Sciences was reported at 80% after the third peak of Covid‐19. In the field of infection prevention and control, the performance score of these hospitals was 96% (good), which is in line with the present study.^13^ One of the important issues in disaster preparation is the issue of staff and equipment safety during disasters, so that the personnel should be familiar with safety issues and instructions and also receive the necessary training continuously.^13^

Resource management, including human resources is one of the most important components of preparation and performance in response to Covid‐19 pandemic.^7^ The selected hospitals scored 92 out of 100 in terms of resource management and were classified at a good level. This result is also in line with the studies of Nasiripour et al.^14^ and Amerion et al.^9^ Since resource management, especially human resource management, plays an important role in hospital performance and prevention of resource wastage, planning to prevent resource wastage is necessary, and the crisis management team should have full knowledge of the duties, instructions, organizational charts, and responsibilities.

A study evaluated the psychological preparedness of emergency staff in the Khatam Al‐Anbiya Hospital in Zahedan in response to Covid‐19 and reported the staff's psychological preparedness at an acceptable level. In the present study, psychological services, which have been mentioned in the diagnosis and treatment domain, were also evaluated at a good level, which is in line with the results of the above study.^15^

In a study conducted on the effective strategies to prevent Covid‐19 disease in Taiwan's KCG Hospital,^16^ solutions including quick and early identification of suspicious cases, quick implementation of preventive or containment measures, the role of training employees and physicians, and human resource management were identified, which are somewhat consistent with the items raised in the checklist of the present study.

In the study of Khorsand Choubdar and Rahdar, which was conducted on the preparedness of hospitals in Sistan and Baluchistan province of Iran, facilitating communication obtained the lowest score among other areas of hospital preparation, which is contrary to the results of the present study.^17^ Ambat assessed the preparedness of Indian private hospitals in response to emerging infectious diseases and showed gaps in the implementation of various programs and protocols such as staff training, risk communication, capacity building, laboratory capacity, and infection control.^18^ In the current study, the domain of coordination and communication also received a low score, which should be considered by related officials.

Tiruneh and colleagues conducted a study on the level of preparedness for Covid‐19 pandemic in the hospitals of northwestern Ethiopia, and showed that only one hospital out of eight had an acceptable level of preparedness.^19^ Other studies also show that in low‐income countries, the lack of equipment for diagnosis and management of Covid‐19 pandemic is a challenge, and also the preparation of hospitals is poor.^20^, ^21^

As the lack of performance in the area of coordination and communication was identified, efforts were made to strengthen this area. The absence of a memorandum of understanding between hospitals and forensic medical centers was among the cases of weak performance, so its strengthening was emphasized after the initial evaluation. Also, according to the results, the use of tele‐information and communication methods was recommended to the families of patients and employees, and drafts of key messages to different parties (such as patients, employees, and society) were written about different scenarios related to Covid‐19.

## 5 CONCLUSION

Pandemics have been and are considered as one of the main risks of international communities, and in addition to maintaining preparedness, an effective response is required to reduce human and financial costs. Among the many institutions responsible for the crisis management of unexpected events, the most important role is played by medical centers, so providing preparedness plans and strategies to deal with the crisis will have significant effects on reducing casualties. However, it should be noted that these plans and strategies will only have a positive effect when these crises are followed up for a short period of time, and also the design and formation of this system is based on this fact.

Evaluating the performance of hospitals in response to Covid‐19 pandemic can be helpful in determining the strengths and weaknesses. It can also help hospital managers and health system policy and decision makers to make necessary plans for future crisis. Despite the appropriate performance of selected hospitals in response to Covid‐19 pandemic, it should be noted that this pandemic may last for many months to come, and the optimal performance of hospitals can decrease at any time. Therefore, continuous evaluation of hospitals' performance is recommended.

### 5.1 Limitations

Limitations in data collection were due to the prevailing situation in hospitals during the Covid‐19 pandemic, which made it difficult to collect information due to the busy schedule of employees. There was also the possibility of contamination for the information collectors. This problem was solved by trying to use proper self‐protection methods.

### 5.2 Recommendation

It is suggested that different hospitals use this tool to evaluate and improve their performance. Also, the indicators presented in this tool can be used by hospital managers and employees as a guide for developing future risk management plans for accidents and biological disasters.

## AUTHOR CONTRIBUTIONS

**Simintaj Sharififar**: Data curation; methodology; validation. **Maryam Moradi**: Investigation; writing—original draft; writing—review & editing.

## CONFLICT OF INTEREST STATEMENT

The authors declare that they have no competing interests. All authors have read and approved the final version of the manuscript had full access to all of the data in this study and takes complete responsibility for the integrity of the data and the accuracy of the data analysis.

## TRANSPARENCY STATEMENT

The lead author Maryam Moradi affirms that this manuscript is an honest, accurate, and transparent account of the study being reported; that no important aspects of the study have been omitted; and that any discrepancies from the study as planned (and, if relevant, registered) have been explained.

## Data Availability

any researcher is interested, for a valid reason, they may contact the corresponding author (M. M.), Moradi21922@yahoo.com.
